# Enhancing HCC Treatment: innovatively combining HDAC2 inhibitor with PD-1/PD-L1 inhibition

**DOI:** 10.1186/s12935-023-03051-0

**Published:** 2023-09-16

**Authors:** Rui Han, Changquan Ling, Yuqian Wang, Lingeng Lu

**Affiliations:** 1 Department of Chinese Medicine Oncology, The First Affiliated Hospital of Naval Medical University, Shanghai, 200433 P. R. China; 2 Department of Chinese Medicine, Naval Medical University, Shanghai, 200433 P. R. China; 3 Department of Oncology, The First Affiliated Hospital of Guangzhou University of Chinese Medicine, Guangzhou, 510405 P. R. China; 4 Department of Chronic Disease Epidemiology, Yale School of Public Health, Yale University, 60 College Street, New Haven, CT 06520-8034 USA; 5 School of Medicine, Center for Biomedical Data Science, Yale University, 60 College Street, New Haven, CT 06520-8034 USA; 6 Yale Cancer Center, Yale University, 60 College Street, New Haven, CT 06520-8034 USA

**Keywords:** HCC, HDAC inhibitor, Immune therapy, Oncotherapy sensitizer, PD-L1 nuclear translocation

## Abstract

Hepatocellular carcinoma (HCC) is a malignancy with high morbidity and mortality but lacks effective treatments thus far. Although the emergence of immune checkpoint inhibitors in recent years has shed light on the treatment of HCC, a considerable number of patients are still unable to achieve durable and ideal clinical benefits. Therefore, refining the combination of immune checkpoint inhibitors (ICIs) to enhance the therapeutic effect has become a global research hotspot. Several histone deacetylase 2 inhibitors have shown advantages in ICIs in many solid cancers, except for HCC. Additionally, the latest evidence has shown that histone deacetylase 2 inhibition can regulate PD-L1 acetylation, thereby blocking the nuclear translocation of PD-L1 and consequently enhancing the efficacy of PD-1/PD-L1 inhibitors and improving anti-cancer immunity. Moreover, our team has recently discovered a novel HDAC2 inhibitor (HDAC2i), valetric acid (VA), that possesses great potential in HCC treatment as a monotherapy. Thus, a new combination strategy, combining HDAC2 inhibitors with ICIs, has emerged with significant development value. This perspective aims to ignite enthusiasm for exploring the application of ideal HDAC2 inhibitors with solid anti-tumor efficacy in combination with immunotherapy for HCC.

## Introduction

Hepatocellular carcinoma (HCC) stands as the predominant form of liver cancer, accounting for nearly 85% of cases. It ranks as the third-most common malignancy in terms of occurrence and cancer-related deaths [1, 2]. HCC is the fastest growing cancer type in the United States, and its incidence has tripled during the past 20 years [3]. Patients with an early-stage HCC can be cured by local ablation, surgical resection, or liver transplantation. However, the recurrence rate is still high and the five-year survival rate after surgery is nearly 35% [4]. In addition, more than half of HCC patients are diagnosed at an advanced stage, limiting the available effective therapeutic strategies. Despite significant advancements in HCC treatment in recent years, including the development of multikinase inhibitors (MKIs) such as lenvatinib, cabozantinib, and regorafenib, approved for advanced or metastatic HCC treatment, the options for available therapeutic strategies for these patients remain limited [5–7]. Furthermore, a significant number of HCC patients continue to experience a lack of durable and optimal clinical benefits, even when utilizing these new treatment alternatives [6–9].

HCC is considered an inflammation-related cancer due to its derivation from chronic inflammatory liver damage, such as damage caused by the hepatitis B virus. Consequently, HCC patients are theoretically thought to benefit from immunotherapy [10, 11] [12]. While notable advancements have been made in understanding HCC immunogenicity, clinical trials have shown limited efficacy of immune checkpoint inhibitor (ICI) monotherapy in treating HCC, with only a small number of patients benefiting from treatment [13, 14]. Moreover, combination therapies involving ICIs and other anticancer agents, including multikinase inhibitors, have been evaluated in phase I to III clinical trials for the treatment of unresectable, treatment-naive HCC [5, 14–18]. The results of these trials have demonstrated that ICI-based combination therapies can provide better clinical benefits than monotherapy, as evidenced by the superior overall survival and progression-free survival rates observed in the IMbrave150 trial and the HIMALAYA trial [19] [20]. However, several unanswered questions remain, such as the lack of biomarkers predictive of response to immunotherapy and the existence of a proportion of patients who do not benefit from ICIs [17, 21, 22]. Therefore, exploring novel and effective ICI-based combination therapeutic strategies to obtain better clinical benefits remains a challenging and important issue in the field of HCC treatment [23–27].

Recently, it has been reported that by interfering histone deacetylases 2 (HDAC2), the acetylation-dependent nuclear translocation of PD-L1 was blocked, thereby reprogramming immune-response-related gene expression and leading to enhanced antitumor response to PD-1 blockades. Meanwhile, HDAC2i (HDAC2 inhibitor) has also been reported to play a crucial role in regulating the expression of PD-L1 induced by interferon-γ (IFN-γ) [28–31]. Thus, the combination of HDAC2 inhibitor and PD-1/PD-L1 immunotherapy is hypothesized as a novel tumor treatment strategy with great clinical application and research prospects, which provides a new opportunity to further improve the overall prognosis of patients with HCC [28–31].

## Progresses of ICI application in HCC treatment

While previous studies have shown that single-agent ICIs have limited efficacy in improving patients diagnosed with HCC, the combination therapy strategy of ICIs has been developed to enhance the clinical benefits for patients. Combination therapy with PD-1 and CTLA-4 inhibitors has demonstrated significantly enhanced efficacy in the treatment of melanoma and HCC patients compared to either agent alone [32]. This is believed to occur due to the inhibition of PD-1/PD-L1, which can reinvigorate exhausted CD8 + T cells, enhance the ability of APCs to present TAA or neoantigens, and promote effector lymphocyte infiltration in tumor tissue. Additionally, since PD-1 and CTLA-4 are expressed on CD8 + T cells and Treg, respectively, as well as other immune cells at different levels, this combination therapy can indirectly improve the tumor immune microenvironment and further enhance the anti-tumor effects [32]. According to data presented by the American Society of Clinical Oncology (ASCO) in 2019, patients with resectable liver cancer achieved complete remission in 29% of cases when treated with nivolumab combined with CTLA-4 inhibitor (ipilimumab), while patients with unresectable liver cancer achieved an objective response rate of up to 25% [33].

**Fig. 1:**
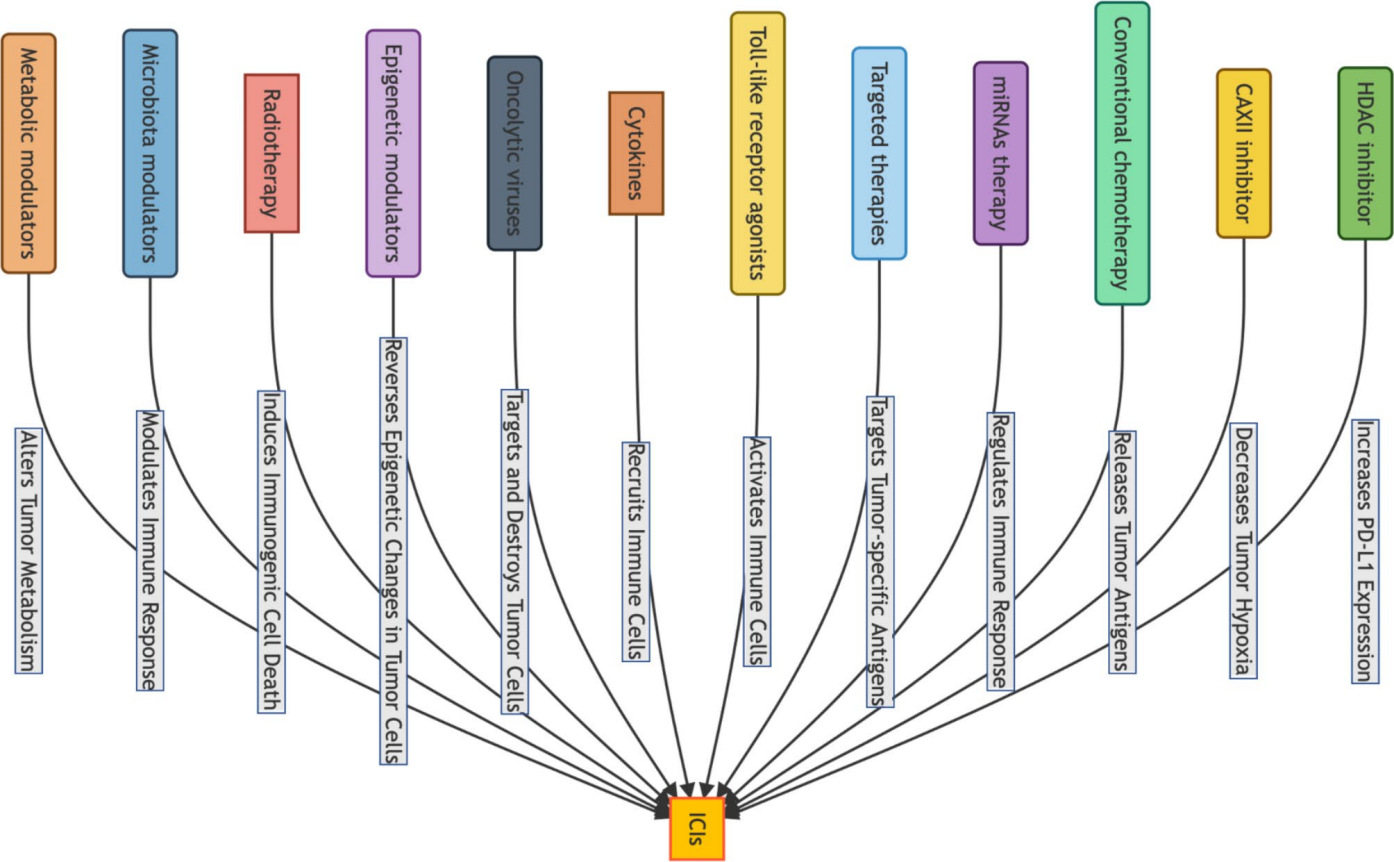
The different therapeutic approaches which can improve the therapeutic effect of ICIs base therapy, as potential sensitizer, in HCC treatment

In addition, though tyrosine kinase inhibitors (TKIs) used in targeted therapy have been effective in clinical trials, they have limited utility for HCC patients due to drug resistance and adverse effects. By combining ICIs and targeted therapy, a new therapeutic approach has been developed that provides greater clinical benefits. In addition to the Pembrolizumab and Lenvatinib combination, other evidence suggests that MET inhibitors, such as tivantinib and capmatinib, can increase PD-L1 expression [34, 35]. In combination with PD-1/PD-L1 inhibitors, the MET inhibitors can significantly inhibit tumor growth and prolong the survival of patients with advanced HCC [36]. Moreover, in patients with advanced HCC resistant to sorafenib, regorafenib followed by the sequential use of PD-1/PD-L1 inhibitors can benefit patients’ survival [37]. More clinical trials of ICIs and TKIs in combination for the treatment of advanced HCC are still ongoing [38]. As adjuvants, ICIs are applied in the clinical management of HCC. However, better ICIs-based therapeutic strategies are still being explored [4]. Furthermore, there are some additional potential sensitizers have been thought to improve the overall response rates and survival outcomes for patients with HCC, overcoming the limitations of ICIs as a monotherapy, such as histone deacetylases inhibitor (HDAC inhibitor), microRNAs (miRNAs) therapy, Toll-like receptor agonists, cytokines and oncolytic viruses, etc [39–45] [46, 47] (Fig. 1). The ideal sensitizer has been considered to possess a synergistic effect with ICIs, modulate the tumor microenvironment to promote an immune response, and have a favorable safety profile, which making HDAC2 inhibitor a proper candidate [44, 48].

## Current status of HDAC inhibitor monotherapy and combined therapy in cancer treatment

Histone deacetylases (HDACs) are a family of deacetylation proteases. There are 18 mammalian HDACs in such family, which are divided into four categories based on the homology between HDACs and yeast HDACs: class I HDACs (HDAC1, -2, -3, and − 8); class II HDACs (HDAC4, -5, -6, -7, -9, and − 10); class III HDACs (sirt1–7); and class IV HDAC (HDAC11) [49]. They can remove the acetyl functional group from the lysine residues at the N-terminus of histones, causing chromatin to bend, allowing DNA to wrap more tightly around histones, which in turn blocks the process of gene transcription. In cancer cells, the overexpression of HDACs can lead to an abnormal increase in their own activity, thereby promoting decreased acetylation and leads to an imbalance in the expression of certain molecules that affect cell proliferation and regulate the cell cycle, including blocking the expression of some tumor suppressor genes (including p53) [50].

Agents that can specifically inhibit HDACs, namely as histone deacetylase inhibitors, have been developed for increasing the efficacy of anti-cancer therapy. Class I HDAC inhibitor is a certain type that can exert inhibitory effect on HDAC2. For instance, the class I HDAC inhibitor SAHA (Vorinostat) was approved by the U.S. FDA in 2006 for the treatment of cutaneous T-lymphocyte tumors, the class I HDAC inhibitor Mocetinostat was for bladder cancer in 2014, and Panobinostat (class I HDAC inhibitor) was for multiple myeloma [50]. Moreover, as a phase 1/II trial shown, for lung cancer treatment, benefit has been provided by the combination therapy of HDAC inhibitor entinostat with 5-AZA-dC, highlighting the potential for HDAC inhibitor to enhance response to other epigenetic therapies [51]. In an animal study, HDAC inhibitor, vorinostat, has also been reported to augment CD8 T-cell infiltration in human lung tumors [52]. For HCC, Panobinostat (HDAC inhibitor) has been found to improve the radiosensitization effect by affecting NKG2D-dependent natural killer cytotoxicity in animal models [53]. In the preclinical setting, the function of immune modulation possessed by HDAC inhibitor has been displayed by growing evidence [52–56]. For instance, the combined inhibition of HDAC1/2 and epigenetic reader Brd4, has been observed to decrease the aberrant IFN-stimulated gene (ISG) expression, thereby modulating the innate and adaptive immune systems, in animal models [57]. In pancreatic cancer cell models, a class I and II HDAC inhibitor, Trichostatin A, has been found to suppress the transcriptional activity of STAT1 by blocking IFN-γinduced STAT1 phosphorylation [33]. Similarly, inhibition of HDAC1 and 2, was demonstrated to increase the release of GM-CSF (Granulocyte-macrophage colony-stimulating factor), thereby increasing the immune response in animal studies [58]. Based on these findings, class I HDAC inhibitor-based combination therapeutic approaches have been developed for treating solid tumors. For instance, Vorinostat (HDAC inhibitor) combined with targeted drug, Olaparib, have been tested in a phase I study (NCT03742245) for patients with relapsed/refractory and/or metastatic breast cancer [59]. Another phase II study is evaluating the efficacy of chidamide (HDAC inhibitor) combined with chemotherapy in neoadjuvant treatment of HR+/HER2- breast cancer (NCT05400993) [59]. In addition, Entinostat (HDAC inhibitor) and Zen003694, as a new anti-cancer drug combination, their application in patients with advanced and refractory solid tumors and Lymphomas, has also been under assessing in a phase II clinical trial (NCT05053971) [60]. Furthermore, a clinical trial that is presently in the recruitment phase seeks to assess the safety, tolerability, and preliminary clinical activity of HG146 (HDACi) when administered orally alone, or when co-administered with a PD-L1 inhibitor, in subjects dealing with refractory/relapsed solid tumors or lymphoma (NCT04977167) [61]. Additionally, another Phase II trial is underway to evaluate the efficacy of nanatinostat (HDACi) when used in combination with valganciclovir among patients with relapsed/refractory EBV-positive lymphomas (NCT05011058) [62]. Moreover, the safety and effectiveness of Vorinostat (HDACi) combined with chemoradiation, have being tested in a phase II study (NCT05608369) involving patients with locally advanced HPV-negative head and neck squamous cell carcinoma. A novel anti-cancer drug combination composed of Entinostat (HDACi), nivolumab, and montanide (R) ISA-51 VG is also being evaluated in a Phase I/II clinical trial for its potential application in patients coping with locally advanced esophageal cancer (NCT05898828). It can be seen that the application of HDAC inhibitors possess high flexibility in different strategies for treating cancer.

## Application of HDAC2 inhibitors in combination with PD-1/PD-L1 inhibitors for HCC treatment

As mentioned before, even though immunotherapy has demonstrated its huge potential in cancer treatment, there still remains a significant percentage of patients who are not able to gain clinical benefits even with combinatorial approaches, such as combining a CTLA-4 inhibitor with a PD-1 inhibitor [61]. Thus, an HDAC inhibitor was selected as a single agent class capable of inducing the expression of chemokines, including Ccl5, Cxcl9, and Cxcl10, to potentially enhance T-cell recruitment and increase patients’ response to immunotherapy. This was achieved by screening a library of all FDA-approved oncology drugs [62]. In addition, romidepsin (an HDAC2 inhibitor) has been found to induce a strong T-cell-dependent antitumor response and enhance the therapeutic effect of PD-1 inhibitors on HCC [62].

Therefore, the combination therapy of HDAC inhibitor plus ICIs has been considered a promising approach for treating solid cancers. Several clinical trials have already been carried out to evaluate such a combined regimen in cancer treatment, including HCC(Fig. 1) [62]. For instance, a phase I/II study (NCT05320640) is testing the combination therapy of a PD-1 inhibitor plus HDAC inhibitor in patients with advanced HCC (or other types of advanced cancer) [63] (Table 1). Additionally, the application of the novel therapeutic strategy of Chidamide (an HDAC2i) plus a PD-1 inhibitor in patients with unresectable, recurrent, or metastatic HCC is also being evaluated in a phase II clinical trial (NCT05163483) [64] (Table 1). However, no trial has been performed to specifically evaluate this strategy in HCC treatment so far, which leaves a significant gap waiting to be explored.

**Table 1:** Selected ongoing trials of ICI combined therapy for Solid cancer

Condition or disease	Therapeutic regimen	Targets	Study	Phase	Total patient number	Recruitment Status	Primary Outcome Measures	Secondary Outcome Measures	Line of therapy
Relapsed/Refractory Non-Hodgkin lymphoma; Advanced solid tumors	Chidamide (HDACi) + Immune checkpoint inhibitors (anti-PD1/PD-L1/CTLA4 antibodies) + Decitabine (DNA methyltransferase inhibiotr)	HDAC1,2,3,10	NCT05320640 [87]	I/II	100 (estimated enrollment)	Recruiting	ORR; AEs	DOR; PFS	Subsequent-line
Cervical Cancer; Cervix Cancer; Cervix Neoplasm	Toripalimab (PD-1 inhibitor) + Chidamide (HDACi)	HDAC1,2,3,10	NCT04651127 [62]	I/II	40 (estimated enrollment)	Recruiting	Safety and tolerability; ORR	PFS; DOR; DCR; OS	Subsequent-line
Sophageal squamous cell cancer; Adenocarcinoma of esophagogastric junction; Gastric adenocarcinoma; Unresectable, recurrent or metastatic disease	Chidamide (HDACi) + PD-1 inhibitor (Toripalimab)	HDAC1,2,3,10	NCT05163483 [63]	II	87 (estimated enrollment)	Recruiting	ORR	PFS; DOR; DCR; OS	Subsequent-line
Solid tumors that are metastatic or cannot be removed by surgery or locally advanced or metastatic HER2-negative breast cancer	Entinostat (HDACi) + Nivolumab and/or Ipilimumab	Class I HDAC	NCT02453620 [88]	I	57	Recruiting	AEs	ORR PFS; DOR	Subsequent-line
Advanced or metastatic non-small cell lung cancer with PD-L1 expression of ≥ 1%	HBI-8000 (HDACi) + Pembrolizumab	Class I HDAC	NCT05141357 [89]	II	24 (estimated enrollment)	Active, not recruiting	ORR	DOR; DCR; PFS; Safety and tolerability	Subsequent-line (No prior treatment with checkpoint inhibitors or more than one regimen of chemotherapy including epidermal growth factor receptor (EGFR) or anaplastic lymphoma kinase (ALK) mutation directed therapy for advanced or metastatic disease)
NSCLC; Melanoma; Mismatch-repair proficient colorectal cancer	Entinostat + Pembrolizumab	Class I HDAC	NCT02437136 [90]	I/II	202	Recruiting	Safety and Tolerability; AEs; ORR	CBR; PFS; OS; DOR; TTR	Subsequent-line
Children and adolescents with high risk refractory/relapsed/progressive tumors (CNS Tumor , Solid Tumor)	Entinostat + Nivolumab	Class I HDAC	NCT03838042 [91]	I/II	128 (estimated enrollment)	Recruiting	DLT; Best response (CR or PR)	DOR; DCR; SD; PFS, etc.	Subsequent-line

Abbreviation: HDACi, HDAC inhibitor; CNS tumors, central nervous system tumors; NSCLC, non-small cell lung cancer; ORR, objective response rate; OS, overall survival; PFS, progression free survival; DOR, duration of response; DCR, disease control rate; DLTs, incidence of dose limiting toxicities; AEs, incidence of adverse events; CBR, clinical benefit rate; TTR, time to response; DLT, dose limiting toxicity; RECIST 1.1, response evaluation criteria in solid tumors 1.1

Moreover, recent evidence has further proven that HDAC2 inhibition can significantly increase the therapeutic effects of PD-1 inhibitors in HCC by suppressing PD-L1 nuclear translocation in animal models. This emphasizes the immense potential of such a combined therapy in HCC treatment [65].

### PD-L1 nuclear translocation causes tumor resistance to immunotherapy

Immunotherapy targeting programmed cell death protein 1 (PD-1) and its ligand PD-L1, as well as cytotoxic T lymphocyte-associated protein 4 (CTLA-4) has shown impressive clinical benefits against a variety of cancer types. However, not all patients achieve durable responses and are resistant to PD-1/PD-L1 inhibitors. The nuclear translocation of PD-L1 has been thought to be an important factor hindering its therapeutic effect [28, 66]. PD-L1 was found not only to have immunosuppressive functions on the plasma membrane, but also to improve the anti-apoptotic ability, promote mTOR activity and regulate glycolytic metabolism of cancer cells [28, 67]. Recent studies have found that PD-L1 can further translocate into the nucleus to modulate pro-inflammatory and immune responses, promote tumor invasiveness and distant metastasis, thereby enabling cancer cells to gain immunotherapy resistance [66].

### PD-L1 nuclear translocation and anti-cancer immunity related to HDAC2

Growing evidence has displayed the immunoregulation capability possessed by the therapy of HDAC inhibition [48]. For instance, HDAC inhibition has been reported to promote the infiltration of CD8 + and CD4 + T cells, and re-polarize tumor-associated macrophages into proinflammatory M1, therefore enhancing the antitumor immune responses and weakening the immune suppression [68]. Suppression of HDACs has also been found to enhance the long-term antitumor immunity and inhibit the development of cancer cells by restoring IFN signaling [69]. HDAC suppression can also restore the B-cell functions by regulating the process of suppressing excessive CTLA4 induction in protein immunization-elicited T cells [70]. Evidence has further reported that HDACs suppression can positively improve tumor microenvironment, by promoting the expression of antigen-presenting machinery genes and CTL infiltration, enhancing T cell activation, suppressing the polarization of M2 macrophages and the trafficking of myeloid-derived suppressor cells, eventually enhancing immunotherapy response, such as promoting the antitumor effect of PD-1/PD-L1 inhibitors, in HCC cells [28, 71].

HDAC2, the most important deacetylases involved in epigenetic regulation, has been found to be correlated with tumor epithelial-to-mesenchymal transition (EMT) process, tumor metastasis, higher Ki-67 level, and multidrug resistance protein expression in cancer cells [72–75]. It also plays an important role in immune defense by regulating the expression of inflammatory genes via the c-Jun/PAI-1 pathway [76]. Moreover, HDAC2 inhibition can also regulate the development of T-cell, thereby affecting cytokine signaling involved in immune response [77]. In breast cancer cells, HDAC2 has also been reported to regulate immune evasion and hold the potential to exacerbate immunosuppression by upregulating tumor PD-L1 expression, suggesting that HDAC2 is a promising therapeutic target to control tumor progression [30]. Furthermore, it has been proved that HDAC2 can modulate the immune microenvironment via regulating the expression of IL-17 A and IL-6, which can recruit myelogenous suppressor cells (MDSC) to inhibit anti-tumor immunity, and promote tumor progression [78–80]. In addition, IL-17 A has been proved to promote CXCR2‑dependent angiogenesis of liver cancer cells [81].

More importantly, recent evidence reported the phenomenon that HDAC2-related deacetylation-dependent nuclear translocation of PD-L1 promotes tumor immune evasion [28]. The nuclear translocation of PD-L1 from the plasma membrane occurs after PD-L1 interacts with the components of endocytosis and nucleocytoplasmic transport-related pathways. This process is regulated by the level of acetylation at Lys 263 in the C-tail of PD-L1, which is controlled by HDAC2 [28–31]. Furthermore, Huntingtin-interacting protein 1-related (HIP1R) can specifically interact with the PD-L1 C tail to initiate the nuclear translocation of PD-L1, and which is regulated by adaptin-β2 (AP2B1). HIP1R can also act as a bridging protein through the adaptor protein AP2B1 to tether unacetylated PD-L1 to clathrin for endocytosis. Furthermore, the expression of HIP1R and its binding ability to PD-L1 are affected by the degree of acetylation of Lys 263 with the hyperacetylation blocking the binding of HIP1R to PD-L1 [28, 82]. In addition, once the HDAC2-mediated deacetylation of PD-L1 occurs, other key regulatory proteins are recruited and activate the PD-L1 endocytosis and nuclear translocation. For instance, vimentin (VIM), as a cytoskeleton protein, can subsequently interact with deacetylated PD-L1 on the plasma membrane, as a carrier eventually carry them into the nucleus under the assistance of importin-α1(KPNA2) [28]. Consequently, accumulated nuclear PD-L1 can further transactivate the immune responsive in the nucleus, leading to the evasion of immune surveillance during metastasis, and the tumor resistance to PD-1 blockage treatment, by regulating the expression levels of various immune checkpoint gene (such as, PD-L2, B7-H3 and VISTA) in HCC cells [28]. Hence, HDAC2 inhibitor has been considered as a sensitizer for PD-1/PD-L1 inhibitors by reducing PD-L1 nuclear localization.

Moreover, the combination of PD-1 inhibitor with HDAC2 inhibitor was found to significantly increase the proportion of CD8 + and CD8 + Gran B (granzyme B) + T cells in tumor-infiltrating lymphocytes (TIL), as well as the ratio of CD8 + cytotoxic T cells and regulatory T cells (CD4 + FOXP3+) [28]. Furthermore, the intervention of HDAC inhibitors can further modulate the levels of cytokines, including IL-4, IFN-γ and TNF-α. TNF-α blockage could overcome resistance to immunotherapy. Intriguingly, anti-PD-1 combined with anti-TNF- α agent has also been reported as a novel therapeutic strategy [28]. An ongoing phase Ib clinical trial (NCT03293784) evaluates the safety and tolerability of the combination in patients with metastatic melanoma [83].

Clearly, the effects of HDAC2 inhibitor on PD-L1 nuclear translocation can interfere with the tumor microenvironment (TME), including affecting the expression of downstream immune-related genes (STAT3, p65, c-Jun40, VSIR, etc.), and cytokine levels (such as reducing TNF-alpha level). It can also enhance the anti-tumor effect of PD-1 inhibitor, which further proves the feasibility and great potential of such a therapeutic strategy, and warrants more related research attempts [28, 83].

### HDAC2 inhibition blocking IFN-γ induced PD-L1 expression

Recent evidence has newly discovered that HDAC2i can regulate IFN-γ induced PD-L1 expression and tumor immune evasion [30]. Briefly, in triple-negative breast cancer (TNBC) cells, HDAC2 has been found to play a pivotal role in promoting the induction of PD-L1 by enhancing the phosphorylation of JAK1 (Januskinase 1), JAK2 (Januskinase 1) and STAT1 (signal transducer and activator of transcription). This effect was accompanied by the translocation of STAT1 into the nucleus, as well as the recruitment of STAT1 to the PD-L1 promoter. Simultaneously, STAT1 facilitated the recruitment of HDAC2 to the PD-L1 promoter. Notably, the elimination of HDAC2 resulted in a compromised IFN-γ-triggered enhancement of H3K27 (Acetylated H3 lysine 27) and H3K9 (Acetylated H3 lysine 9) acetylation, along with diminished recruitment of BRD4 (bromodomain containing 4) to the PD-L1 promoter [28]. Moreover, the in vitro knockout of HDAC2 led to a significant reduction in the proliferation, colony formation, migration, and disruption of the cell cycle in triple-negative breast cancer (TNBC) cells. Additionally, the absence of HDAC2 diminished the IFN-γ-induced expression of PD-L1, curtailed lymphocyte infiltration, and impeded tumor growth and metastasis in breast cancer mouse models [30] [84]. However, it should be noted that whether these identical mechanisms apply to HCC cells necessitates further investigation.

### Selection of potential HDAC2 inhibitor

**Fig. 2:**
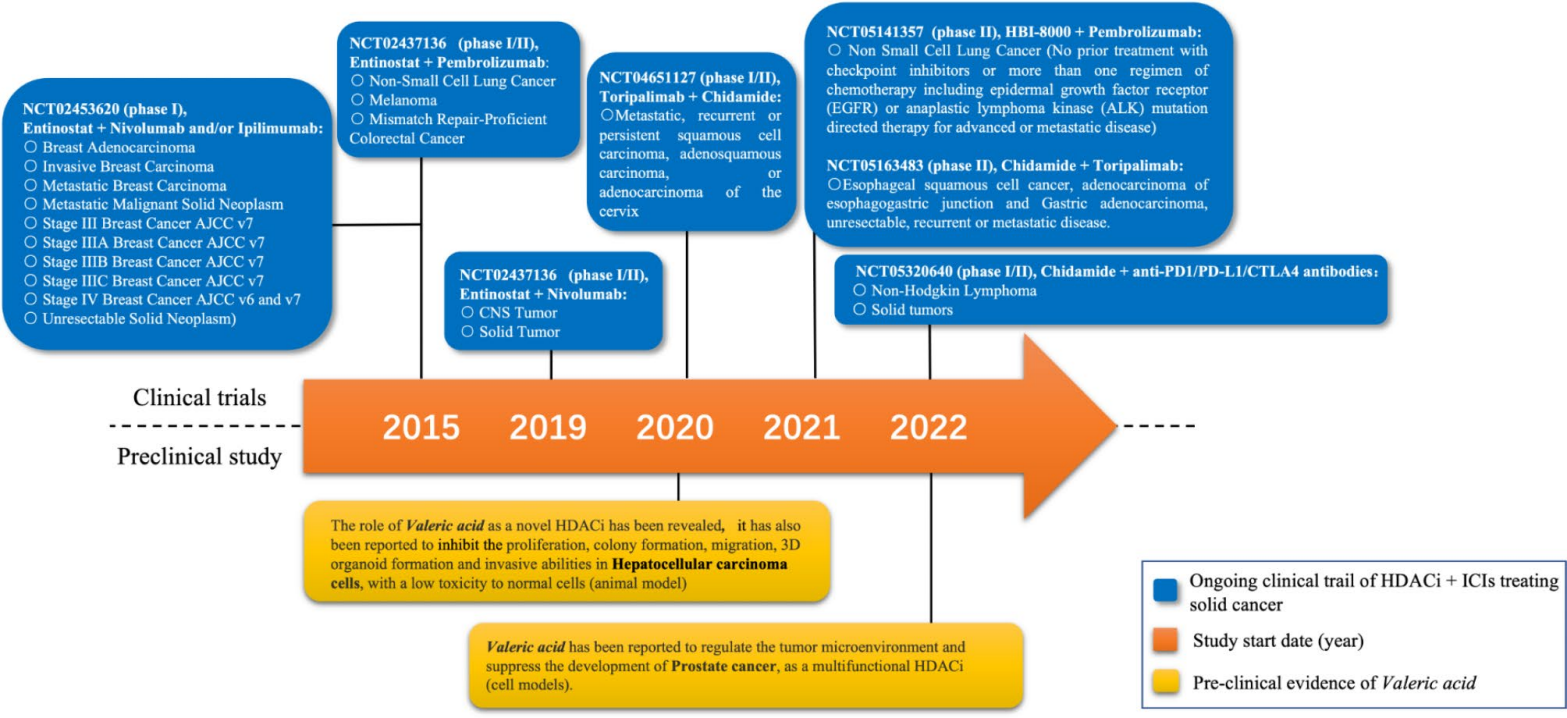
Milestone chart on the progress of selected HDAC inhibitors research. There is currently no clinical trial specifically targeting the treatment of hepatocellular carcinoma (HCC) with HDAC inhibitor. However, VA monotherapy has been found to inhibit the development of HCC cells by preclinical study

It is crucial to choose an appropriate HDAC inhibitor. Some HDAC2 inhibitors have already been reported to possess excellent anti-cancer effects. Therefore, it would be more intriguing if the anti-cancer effect could be further enhanced by combination therapies. Previous studies conducted by our group have provided us a potential candidate of HDAC inhibitor, Valeric Acid (VA) [85–87] (Fig. 2).

**Fig. 3:**
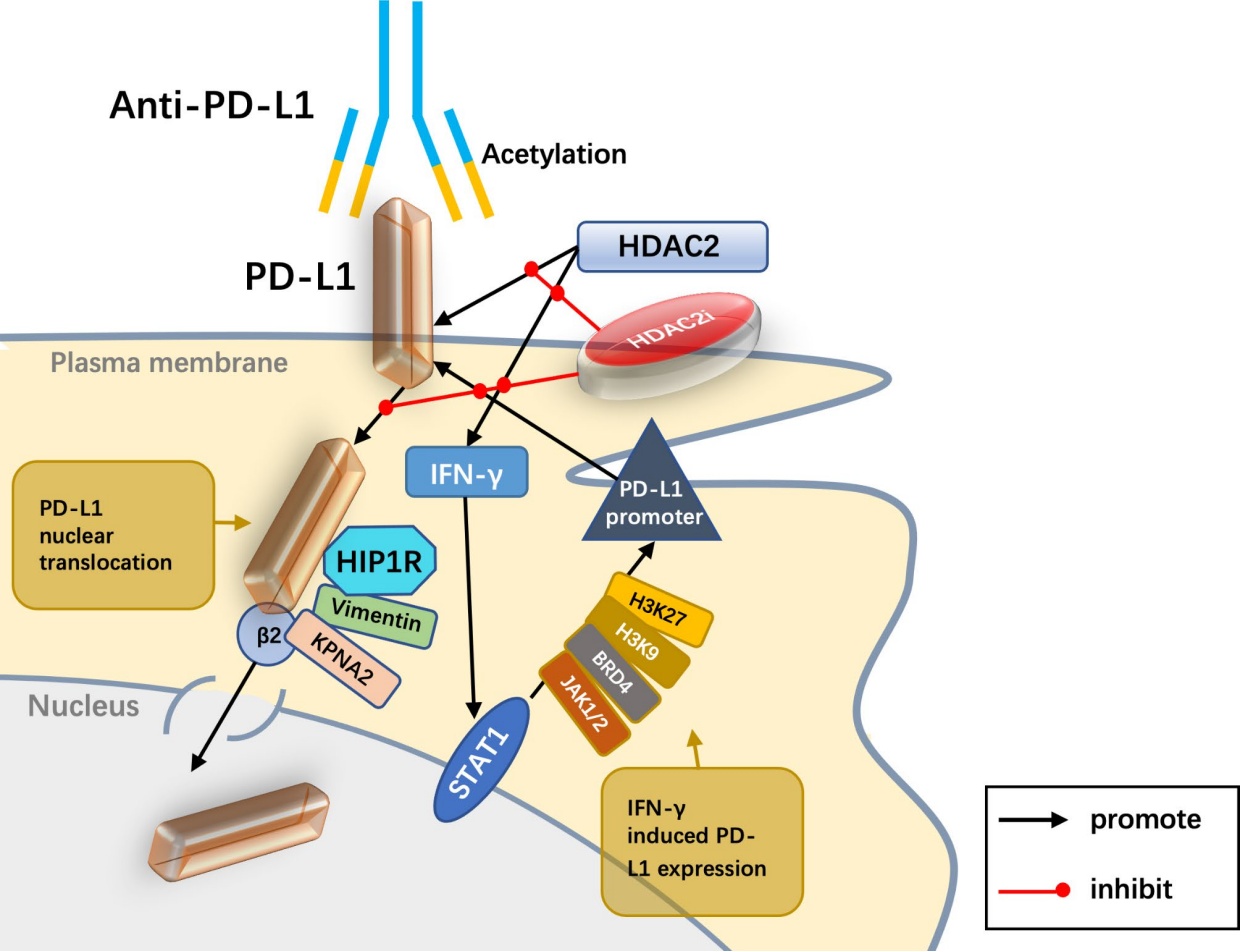
Potential mechanisms of HDAC2i acts as sensitizer for PD-1/PD-L1 blockade therapy. HDAC2i can suppress the PD-L1 nuclear translocation regulated by regulating HIP1R, Vimentin and KPNAS. Moreover, HDAC2i can also inhibit the process of IFNγ-induced PD-L1 expression regulated by STAT1, BRD4, H3K27, etc.

VA is extracted from Valerian (Valeriana officinalis) which is a member of Valerianaceae family. Such an herb has been approved by the US Food and Drug Administration (FDA) as an alternative medicine to treat insomnia and other sleep-related disorders. It is also a drug in Traditional Chinese Medicine which has been applied for liver diseases treatment for thousands of years [88]. VA, as one of the most active components of Valerian, has been found as a typical HDAC2 inhibitor, also a class I HDAC inhibitor, which also possesses low toxicity to normal cells and multiple anti-cancer abilities (Inhibition of proliferation, colony cloning, invasion, migration, 3D organoid formation, etc.) in HCC, prostate cancer and breast cancer cells (both in vitro and in vivo) [85–87]. Moreover, our studies also revealed its regulatory effects on inhibiting tumor stemness and regulating apoptosis, etc. Moreover, we screened and refined a suitable lipid based nanoparticle (LNP-DP1) for liver-targeted delivery in HCC cancer with higher therapeutic effect on the tumor cells but lower toxicity to normal cells, in animal study [85]. This further adds liver targeting property of VA compared to other HDAC2 inhibitors. Therefore, the effect of VA on the efficacy of PD-1 antibody becomes more intriguing. The potential mechanisms of VA underlying its immunity regulation and enhancing the efficacy of PD-1/PD-L1 immunotherapy are illustrated as Fig. 3.

### Future directions and potential challenges

Both preclinical studies and early-phase clinical trials have demonstrated promising outcomes when combining HDAC inhibitors with immunotherapy for treating solid tumors. However, some notable challenges remain, for instance, there are currently no FDA approved specific inhibitors designed solely for HDAC2. The non-selective targeting of multiple HDACs by many HDAC inhibitors can lead to a lack of selectivity, resulting in significant adverse effects in clinical practice such as thrombocytopenia, fatigue, and diarrhea [89]. Therefore, the selection of proper HDAC2i is a major research direction. Furthermore, recent endeavors involve the application of nanoparticles (NPs) for delivering HDAC inhibitors (HDACi) with innovations [90]. NPs function as carriers for both HDACi and anti-PD-1, safeguarding them from degradation, amplifying their bioavailability, and facilitating targeted delivery to precise cells or tissues [91]. In addition, to augment the therapeutic impact of this approach, an array of innovative compounds known as polypharmacological molecules are being actively researched. These compounds may offer a dual inhibition effect, targeting both HDAC2 and other therapeutic markers commonly utilized in focused treatment strategies. For instance, CUDC-101, presently in phase I trials, represents a pioneering small molecule that concomitantly inhibits HDACs alongside receptor kinases like the epidermal growth factor receptor (EGFR) and human epidermal growth factor receptor 2 (HER2) in cancerous cells [92]. Considering the context-dependent nature of epigenetic regulators and their wide-ranging effects, compounds such as HDAC inhibitors can yield diverse outcomes depending on the specific situation. A more comprehensive comprehension of anti-tumor immune evasion, coupled with an in-depth exploration of the impact of epigenetic regulations, will be essential to enhance the effectiveness of combination strategies involving immunotherapies and HDAC2i. Given the situation-dependent behavior of epigenetic regulators and their broad spectrum of influences, HDAC2 inhibitor can potentially generate a variety of results contingent on the particular circumstances. To bolster the efficacy of combination approaches that encompass ICI and HDAC2i, it will be crucial to gain a deeper understanding of anti-tumor immune evasion and thoroughly investigate the consequences of epigenetic regulations.

## Conclusion

A growing body of evidence suggests that HDAC2-inhibiting drugs such as class I HDAC inhibitor, can act not only as standalone anti-cancer agents but also improve the therapeutic effect of ICIs in various types of solid cancers. However, no attempts have been made to combine ICIs with HDAC inhibitors for HCC treatment yet, to our best knowledge, leaving a significant gap in research.

Additionally, new evidence has shown that HDAC2 inhibitors have anti-cancer properties, including in cases of HCC, making them a promising new drug for treating HCC. Recent research has demonstrated that inhibition of HDAC2 can induce anti-tumor immune regulation. This suppression of HDAC2 not only regulates the level of T lymphocyte subsets and inhibits tumor immune suppression but also enhances the effectiveness of PD-1 inhibitors. Therefore, selecting appropriate HDAC2 inhibitors has undoubtedly become an intriguing topic. For instance, valeric acid, a newly reported HDAC2 inhibitor, has shown high efficacy against tumors with low toxicity to normal cells and holds the potential to be a qualified candidate for combination therapy with PD-1 inhibitors in the treatment of HCC. Furthermore, encapsulating VA in LNP-DP1 can further increase its therapeutic effect and safety in HCC treatment, which guarantees future research into investigating the novel combination of VA plus PD-1 inhibitors. Certainly, further research is needed to identify other promising HDAC2 inhibitors to explore more effective therapeutic strategies and novel targets for treating HCC. Other potential therapeutic combinations, such as ICI plus HDAC2 inhibitor and anti-angiogenic agents, which may offer even better outcomes, could also be evaluated.

## Data Availability

The data that support the findings of this study are openly available in ClinicalTrials.gov at https://clinicaltrials.gov.

## References

[1] Alshuwaykh O et al. *Incidence of hepatocellular carcinoma in chronic hepatitis B virus infection in those not meeting criteria for antiviral therapy*. Hepatol Commun, 2022. 10.1002/hep4.2064 PMC9592790 36004713

[2] Llovet JM (2021). Hepatocellular carcinoma. Nat Rev Dis Primers.

[3] Petrelli F (2022). Hepatocellular carcinoma in patients with nonalcoholic fatty liver disease: a systematic review and meta-analysis: HCC and Steatosis or Steatohepatitis. Neoplasia.

[4] Cao W (2021). Changing profiles of cancer burden worldwide and in China: a secondary analysis of the global cancer statistics 2020. Chin Med J (Engl).

[5] Feng MY, Chan LL, Chan SL (2022). Drug Treatment for Advanced Hepatocellular Carcinoma: First-Line and Beyond. Curr Oncol.

[6] Deng S, Solinas A, Calvisi DF (2021). Cabozantinib for HCC Treatment, from clinical back to experimental models. Front Oncol.

[7] Bornschein J, Schlosser S (2017). [Regorafenib - a revolution in the systemic treatment options of HCC?]. Z Gastroenterol.

[8] Roviello G (2019). Ramucirumab as a second line therapy for advanced HCC: a significant achievement or a wasted opportunity for personalised therapy?. Invest New Drugs.

[9] Cinnamon E, Pikarsky E (2020). Are we ready for targeted therapy combinations in HCC?. Gut.

[10] Campbell C (2021). Risk factors for the development of hepatocellular carcinoma (HCC) in chronic hepatitis B virus (HBV) infection: a systematic review and meta-analysis. J Viral Hepat.

[11] Shen Y (2020). Risk factors for hepatocellular carcinoma (HCC) in the northeast of the United States: results of a case-control study. Cancer Causes Control.

[12] Yuan G (2022). Hepatic tumor stiffness measured by Shear Wave Elastography is Prognostic for HCC Progression following treatment with Anti-PD-1 antibodies plus Lenvatinib: a retrospective analysis of two independent cohorts. Front Immunol.

[13] Casak SJ (2021). FDA approval Summary: Atezolizumab Plus Bevacizumab for the treatment of patients with Advanced Unresectable or Metastatic Hepatocellular Carcinoma. Clin Cancer Res.

[14] Ouyang T, Kan X, Zheng C (2022). Immune Checkpoint inhibitors for Advanced Hepatocellular Carcinoma: Monotherapies and Combined Therapies. Front Oncol.

[15] Finn RS (2020). Phase ib study of Lenvatinib Plus Pembrolizumab in patients with Unresectable Hepatocellular Carcinoma. J Clin Oncol.

[16] Kelley RK (2021). Safety, Efficacy, and Pharmacodynamics of Tremelimumab Plus Durvalumab for patients with Unresectable Hepatocellular Carcinoma: Randomized Expansion of a phase I/II study. J Clin Oncol.

[17] Cheng AL (2022). Updated efficacy and safety data from IMbrave150: Atezolizumab plus bevacizumab vs. sorafenib for unresectable hepatocellular carcinoma. J Hepatol.

[18] Lee MS (2020). Atezolizumab with or without bevacizumab in unresectable hepatocellular carcinoma (GO30140): an open-label, multicentre, phase 1b study. Lancet Oncol.

[19] Finn RS (2020). Atezolizumab plus Bevacizumab in Unresectable Hepatocellular Carcinoma. N Engl J Med.

[20] Abou-Alfa GK (2022). Phase 3 randomized, open-label, multicenter study of tremelimumab (T) and durvalumab (D) as first-line therapy in patients (pts) with unresectable hepatocellular carcinoma (uHCC): HIMALAYA. J Clin Oncol.

[21] Han R (2019). MicroRNA-34a suppresses aggressiveness of hepatocellular carcinoma by modulating E2F1, E2F3, and Caspase-3. Cancer Manag Res.

[22] Rizzo A (2021). Predictive biomarkers for checkpoint inhibitor-based immunotherapy in Hepatocellular Carcinoma: where do we stand?. Front Oncol.

[23] Hercun J (2022). Immune-Mediated Hepatitis during Immune Checkpoint inhibitor cancer immunotherapy: Lessons from Autoimmune Hepatitis and Liver Immunology. Front Immunol.

[24] Zhang Y, et al. Emerging insights on immunotherapy in liver cancer. Antioxid Redox Signal; 2022. 10.1089/ars.2022.0047 35754339

[25] Di Federico A (2022). Atezolizumab-bevacizumab plus Y-90 TARE for the treatment of hepatocellular carcinoma: preclinical rationale and ongoing clinical trials. Expert Opin Investig Drugs.

[26] Rizzo A (2021). First-line immune checkpoint inhibitor-based combinations in unresectable hepatocellular carcinoma: current management and future challenges. Expert Rev Gastroenterol Hepatol.

[27] Rizzo A (2022). Which role for predictors of response to immune checkpoint inhibitors in hepatocellular carcinoma?. Expert Rev Gastroenterol Hepatol.

[28] Gao Y (2020). Acetylation-dependent regulation of PD-L1 nuclear translocation dictates the efficacy of anti-PD-1 immunotherapy. Nat Cell Biol.

[29] Jaccard A, Ho PC (2020). The hidden side of PD-L1. Nat Cell Biol.

[30] Xu P (2021). Histone deacetylase 2 knockout suppresses immune escape of triple-negative breast cancer cells via downregulating PD-L1 expression. Cell Death Dis.

[31] Bertrand F (2017). TNFalpha blockade overcomes resistance to anti-PD-1 in experimental melanoma. Nat Commun.

[32] Fulgenzi CAM et al. *Novel immunotherapy combinations in clinical trials for hepatocellular carcinoma: will they shape the future treatment landscape?* Expert Opin Investig Drugs, 2022: p. 1–11. 10.1080/13543784.2022.2072726 35507361

[33] Pinato DJ (2021). PRIME-HCC: phase ib study of neoadjuvant ipilimumab and nivolumab prior to liver resection for hepatocellular carcinoma. BMC Cancer.

[34] Qin S (2019). A phase II study of the efficacy and safety of the MET inhibitor capmatinib (INC280) in patients with advanced hepatocellular carcinoma. Ther Adv Med Oncol.

[35] Wu ZX et al. *Tivantinib, a c-Met inhibitor in clinical trials, is susceptible to ABCG2-Mediated Drug Resistance*. Cancers (Basel), 2020. 12(1). 10.3390/cancers12010186 PMC7017082 31940916

[36] Li H (2019). MET inhibitors promote Liver Tumor Evasion of the Immune response by stabilizing PDL1. Gastroenterology.

[37] Joerger M (2019). Prolonged tumor response associated with sequential immune checkpoint inhibitor combination treatment and regorafenib in a patient with advanced pretreated hepatocellular carcinoma. J Gastrointest Oncol.

[38] Banstola A, Jeong JH, Yook S (2020). Immunoadjuvants for cancer immunotherapy: a review of recent developments. Acta Biomater.

[39] Han CL et al. *Efficacy and safety of immune checkpoint inhibitors for hepatocellular carcinoma patients with macrovascular invasion or extrahepatic spread: a systematic review and meta-analysis of 54 studies with 6187 hepatocellular carcinoma patients*. Cancer Immunol Immunother, 2023. 10.1007/s00262-023-03390-x PMC10991272 36811662

[40] Xia W et al. *PD-1 inhibitor inducing exosomal miR-34a-5p expression mediates the cross talk between cardiomyocyte and macrophage in immune checkpoint inhibitor-related cardiac dysfunction*. J Immunother Cancer, 2020. 8(2). 10.1136/jitc-2020-001293 PMC7594538 33115945

[41] Jabbari N (2020). Modulation of Immune Checkpoints by Chemotherapy in Human Colorectal Liver Metastases. Cell Rep Med.

[42] Carbone C et al. *Intratumoral injection of TLR9 agonist promotes an immunopermissive microenvironment transition and causes cooperative antitumor activity in combination with anti-PD1 in pancreatic cancer*. J Immunother Cancer, 2021. 9(9). 10.1136/jitc-2021-002876 PMC8420705 34479922

[43] Kim Y (2022). The tri-iodothyronine (T3) level is a prognostic factor for patients with Advanced NSCLC: receiving Immune checkpoint inhibitors and is Associated with Liver Metastasis. Clin Med Insights Oncol.

[44] Giraud J (2021). Hepatocellular Carcinoma Immune Landscape and the potential of immunotherapies. Front Immunol.

[45] Ilyas FZ, Beane JD, Pawlik TM. *The state of Immunotherapy in Hepatobiliary Cancers*. Cells, 2021. 10(8). 10.3390/cells10082096 PMC8393650 34440865

[46] Rossi E et al. *Hepatic radiotherapy in addition to Anti-PD-1 for the treatment of metastatic uveal melanoma patients*. Cancers (Basel), 2023. 15(2). 10.3390/cancers15020493 PMC9857311 36672442

[47] Wu H (2022). Dynamic microbiome and metabolome analyses reveal the interaction between gut microbiota and anti-PD-1 based immunotherapy in hepatocellular carcinoma. Int J Cancer.

[48] Hai R (2021). Characterization of histone deacetylase mechanisms in Cancer Development. Front Oncol.

[49] Burns AM (2022). The HDAC inhibitor CI-994 acts as a molecular memory aid by facilitating synaptic and intracellular communication after learning. Proc Natl Acad Sci U S A.

[50] Natarajan U, Venkatesan T, Rathinavelu A. *Effect of the HDAC inhibitor on histone acetylation and methyltransferases in A2780 ovarian Cancer cells*. Med (Kaunas), 2021. 57(5). 10.3390/medicina57050456 PMC8151761 34066975

[51] Juergens RA (2011). Combination epigenetic therapy has efficacy in patients with refractory advanced non-small cell lung cancer. Cancer Discov.

[52] Ma T (2013). Comparing histone deacetylase inhibitor responses in genetically engineered mouse lung cancer models and a window of opportunity trial in patients with lung cancer. Mol Cancer Ther.

[53] Liu YF (2022). Radiosensitization effect by HDAC inhibition improves NKG2D-dependent natural killer cytotoxicity in hepatocellular carcinoma. Front Oncol.

[54] Vo DD (2009). Enhanced antitumor activity induced by adoptive T-cell transfer and adjunctive use of the histone deacetylase inhibitor LAQ824. Cancer Res.

[55] Christiansen AJ (2011). Eradication of solid tumors using histone deacetylase inhibitors combined with immune-stimulating antibodies. Proc Natl Acad Sci U S A.

[56] Yan MM (2022). [Effect of HDAC inhibitor chidamide on PD-L1 expression in Peripheral T-Cell Lymphoma]. Zhongguo Shi Yan Xue Ye Xue Za Zhi.

[57] Marie IJ, Chang HM, Levy DE (2018). HDAC stimulates gene expression through BRD4 availability in response to IFN and in interferonopathies. J Exp Med.

[58] Gatla HR et al. *Regulation of Chemokines and Cytokines by histone deacetylases and an update on histone decetylase inhibitors in Human Diseases*. Int J Mol Sci, 2019. 20(5). 10.3390/ijms20051110 PMC6429312 30841513

[59] Pramanik SD (2022). Potential of histone deacetylase inhibitors in the control and regulation of prostate, breast and ovarian cancer. Front Chem.

[60] Saleh R (2020). Role of epigenetic modifications in Inhibitory Immune Checkpoints in Cancer Development and Progression. Front Immunol.

[61] Brown LC et al. *Evaluation of tumor microenvironment and biomarkers of immune checkpoint inhibitor response in metastatic renal cell carcinoma*. J Immunother Cancer, 2022. 10(10). 10.1136/jitc-2022-005249 PMC9577926 36252996

[62] Zheng H (2016). HDAC inhibitors enhance T-Cell chemokine expression and augment response to PD-1 immunotherapy in Lung Adenocarcinoma. Clin Cancer Res.

[63] Kalinkova L et al. *Targeting DNA methylation in Leukemia, Myelodysplastic Syndrome, and Lymphoma: a potential Diagnostic, Prognostic, and Therapeutic Tool*. Int J Mol Sci, 2022. 24(1). 10.3390/ijms24010633 PMC9820560 36614080

[64] Sun Y (2022). Therapeutic potential of tucidinostat, a subtype-selective HDAC inhibitor, in cancer treatment. Front Pharmacol.

[65] Blaszczak W (2021). Immune modulation underpins the anti-cancer activity of HDAC inhibitors. Mol Oncol.

[66] Chen H (2022). Functional nanovesicles displaying anti-PD-L1 antibodies for programmed photoimmunotherapy. J Nanobiotechnol.

[67] Chang CH (2015). Metabolic competition in the Tumor Microenvironment is a driver of Cancer Progression. Cell.

[68] Li X (2021). HDAC inhibition potentiates anti-tumor activity of macrophages and enhances anti-PD-L1-mediated tumor suppression. Oncogene.

[69] Owen KL (2020). Prostate cancer cell-intrinsic interferon signaling regulates dormancy and metastatic outgrowth in bone. EMBO Rep.

[70] Li F et al. *T(FH) cells depend on Tcf1-intrinsic HDAC activity to suppress CTLA4 and guard B-cell help function*. Proc Natl Acad Sci U S A, 2021. 118(2). 10.1073/pnas.2014562118 PMC7812797 33372138

[71] Kim YD, Inhibitor HDAC (2020). CG-745, enhances the Anti-Cancer Effect of Anti-PD-1 Immune checkpoint inhibitor by modulation of the Immune Microenvironment. J Cancer.

[72] Huober J (2022). Atezolizumab with Neoadjuvant Anti-Human epidermal growth factor receptor 2 therapy and chemotherapy in human epidermal growth factor receptor 2-Positive early breast Cancer: primary results of the Randomized Phase III IMpassion050 Trial. J Clin Oncol.

[73] Rodriguez M et al. *HDAC inhibitors enhance efficacy of the Oncolytic Adenoviruses Ad∆∆ and Ad-3∆-A20T in pancreatic and triple-negative breast Cancer Models*. Viruses, 2022. 14(5). 10.3390/v14051006 PMC9143155 35632748

[74] Wagner T (2014). Histone deacetylase 2 controls p53 and is a critical factor in tumorigenesis. Biochim Biophys Acta.

[75] Müller BM (2013). Differential expression of histone deacetylases HDAC1, 2 and 3 in human breast cancer–overexpression of HDAC2 and HDAC3 is associated with clinicopathological indicators of disease progression. BMC Cancer.

[76] Fang WF (2018). Histone deacetylase 2 (HDAC2) attenuates lipopolysaccharide (LPS)-induced inflammation by regulating PAI-1 expression. J Inflamm (Lond).

[77] Conte M (2015). HDAC2 deregulation in tumorigenesis is causally connected to repression of immune modulation and defense escape. Oncotarget.

[78] Lai T (2019). HDAC2 attenuates airway inflammation by suppressing IL-17A production in HDM-challenged mice. Am J Physiol Lung Cell Mol Physiol.

[79] Islam MR (2022). Polymorphisms in IL-17A gene and susceptibility of Colorectal Cancer in Bangladeshi Population: a case-control analysis. Cancer Control.

[80] Liu W et al. *IL-17A Promotes the Migration, Invasion and the EMT Process of Lung Cancer Accompanied by NLRP3 Activation* Biomed Res Int, 2022. 2022: p. 7841279. 10.1155/2022/7841279 PMC9637470 36349316

[81] Liu L (2019). IL–17A promotes CXCR2–dependent angiogenesis in a mouse model of liver cancer. Mol Med Rep.

[82] Koh YW et al. *HIP1R expression and its association with PD-1 pathway blockade response in Refractory Advanced NonSmall Cell Lung Cancer: a Gene Set Enrichment Analysis*. J Clin Med, 2020. 9(5). 10.3390/jcm9051425 PMC7291156 32403421

[83] Perez-Ruiz E (2019). Prophylactic TNF blockade uncouples efficacy and toxicity in dual CTLA-4 and PD-1 immunotherapy. Nature.

[84] Fan Z (2022). The generation of PD-L1 and PD-L2 in cancer cells: from nuclear chromatin reorganization to extracellular presentation. Acta Pharm Sin B.

[85] Han R (2020). Valeric acid suppresses Liver Cancer Development by acting as a novel HDAC inhibitor. Mol Ther Oncolytics.

[86] Shi F (2021). Valerian and valeric acid inhibit growth of breast cancer cells possibly by mediating epigenetic modifications. Sci Rep.

[87] Han R (2022). Valeric acid acts as a novel HDAC3 inhibitor against prostate cancer. Med Oncol.

[88] Honma T (2019). Seeds of Centranthus ruber and Valeriana officinalis contain conjugated linolenic acids with reported Antitumor Effects. J Oleo Sci.

[89] Liang T (2023). Targeting histone deacetylases for cancer therapy: Trends and challenges. Acta Pharm Sin B.

[90] Lindemann H (2020). Polysaccharide nanoparticles bearing HDAC inhibitor as nontoxic nanocarrier for drug delivery. Macromol Biosci.

[91] Shen C (2023). HDAC inhibitors enhance the anti-tumor effect of immunotherapies in hepatocellular carcinoma. Front Immunol.

[92] Lai CJ (2010). CUDC-101, a multitargeted inhibitor of histone deacetylase, epidermal growth factor receptor, and human epidermal growth factor receptor 2, exerts potent anticancer activity. Cancer Res.

